# Small Reduced Graphene Oxides for Highly Efficient Oxygen Reduction Catalysts

**DOI:** 10.3390/ijms222212300

**Published:** 2021-11-14

**Authors:** Su-Jeong Bak, Sun-I Kim, Su-yeong Lim, Taehyo Kim, Se-Hun Kwon, Duck Hyun Lee

**Affiliations:** 1 Green Materials and Processes R&D Group, Korea Institute of Industrial Technology, Ulsan 44413, Korea; baksj@kitech.re.kr (S.-J.B.); sunikim@kitech.re.kr (S.-I.K.); swimmingly@kitech.re.kr (S.-y.L.); thkim0215@kitech.re.kr (T.K.); 2 Department of Materials Science & Engineering, Pusan National University, Busan 46241, Korea

**Keywords:** oxygen reduction reaction, proton exchange membrane fuel cell, graphene, Pt catalyst

## Abstract

We demonstrated highly efficient oxygen reduction catalysts composed of uniform Pt nanoparticles on small, reduced graphene oxides (srGO). The reduced graphene oxide (rGO) size was controlled by applying ultrasonication, and the resultant srGO enabled the morphological control of the Pt nanoparticles. The prepared catalysts provided efficient surface reactions and exhibited large surface areas and high metal dispersions. The resulting Pt/srGO samples exhibited excellent oxygen reduction performance and high stability over 1000 cycles of accelerated durability tests, especially the sample treated with 2 h of sonication. Detailed investigations of the structural and electrochemical properties of the resulting catalysts suggested that both the chemical functionality and electrical conductivity of these samples greatly influence their enhanced oxygen reduction efficiency.

## 1. Introduction

Fuel cells are an essential component of highly efficient and clean energy-production technologies. In particular, proton-exchange membrane fuel cells (PEMFCs) have shown great potential for power generation in stationary, mobile, and transportation applications owing to their low temperature, low emissions, rapid start-up time, energy efficiency, and power density [1,2,3]. The cathodic oxygen reduction reaction (ORR) is the rate-limiting reaction step in PEMFCs. Pt has been used as an ORR catalyst for over a decade because of its high catalytic activity [4], and its high cost has been combatted by the use of carbon black (CB), which reduces the required amount of Pt and enhances the stability of PEMFC active materials. However, CB has been shown to have low catalytic durability because it easily corrodes under an oxygen atmosphere [1,3,5]. To improve the long-term durability of PEMFC catalysts, carbon nanomaterials such as carbon nanotubes (CNTs), nanofibers, and graphene have been widely studied as support materials [1,6,7,8]. Graphene has attracted significant attention as a promising catalyst support owing to its unique 2-D structure, high conductivity, and large surface area [9,10,11,12,13,14]. In particular, graphene oxide (GO) is a promising support material that can serve as an electrocatalyst for PEMFCs owing to its abundant surface functional groups, which are chemically active sites that can be used for catalytic reactions and also act as anchoring sites for metal nanoparticles. However, excessive amounts of oxygen-containing functional groups can reduce the electrical conductivity and electrochemical stability of these systems, making them susceptible to chemical oxidation and decreasing their long-term durability [1,15,16,17,18,19]. Of all graphene-related materials, the most widely available and commonly used is reduced GO (rGO). The surface oxygen-containing groups located on the corrugated graphene layers of this material facilitate exfoliation, and its excellent dispersion of metal nanoparticles with a narrow range of sizes allows for its wide application [19,20,21].

Efficient ORR activity and durability can be achieved by combining the large surface area of a catalyst and the mass transfer of reactants on its surface, which is controlled by the morphology of the catalyst [22,23,24]. Therefore, the morphology of the catalyst is the main parameter that can significantly affect catalytic activity, selectivity, and stability. Several studies have shown that smaller catalyst supports result in higher conversions and yields for the ORR [25,26,27,28]. This is because the small sizes of these catalysts result in efficient surface reactions, due to their large surface areas, high metal dispersions, and minimal internal diffusion effects. Therefore, the size of the support and the interactions between the metal and support make an important contribution to maximizing the activity and stability of such catalysts. Herein, we report highly efficient oxygen reduction catalysts composed of uniform Pt nanoparticles on small rGO (srGO). Ultrasonication is used with varying times to control the rGO size, and the srGO enable the morphological control of the Pt nanoparticles. Consequently, the performance and stability of the Pt/srGO samples are enhanced compared to those of Pt/rGO and Pt/CB in an acidic medium. A detailed investigation of the structural and electrochemical proper-ties of these catalysts was conducted to examine the impact of their chemical functionalities and electrical conductivities.

## 2. Results and Discussion

**Figure 1:**
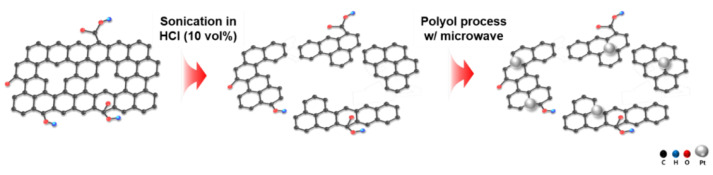
Schematic illustration of the synthesis of Pt/srGO.

Figure 1 shows the synthetic procedure of Pt/srGO. Samples of rGO were mixed with HCl and treated via ultrasonication for 1, 2, or 3 h, and strong sonication was used to break the weak carbon bonds and reduce the size of rGO. Specifically, rGO was dispersed in an acidic solution to weaken the bonds, and then a physical external force was applied to remove the portions of carbon bonds where the bonding force was weak.

**Figure 2:**
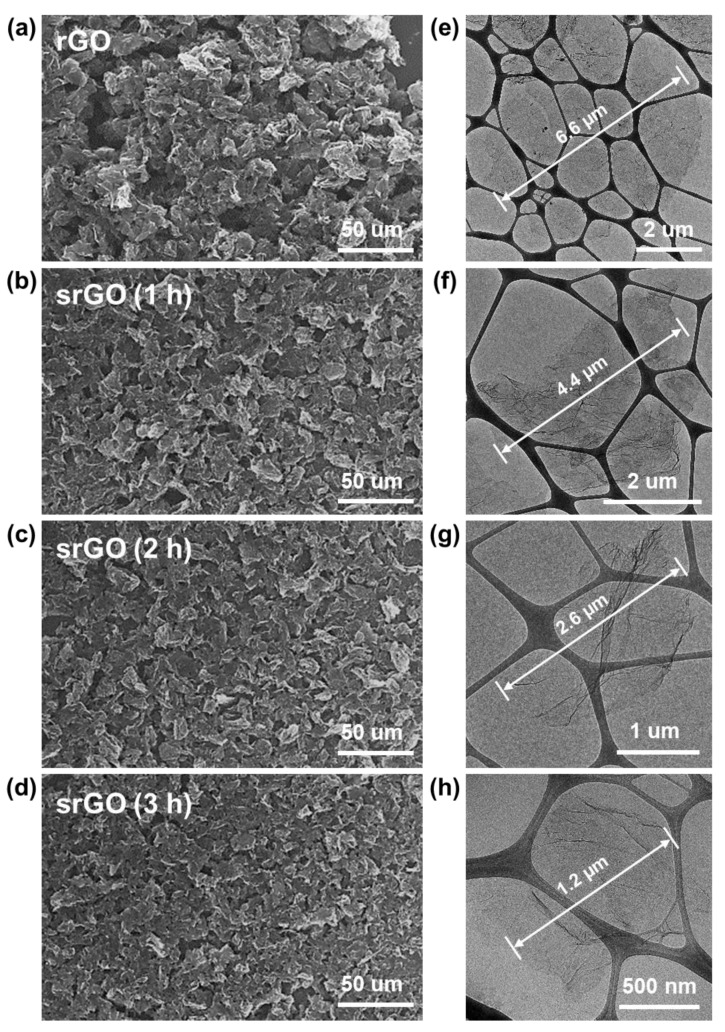
FE-SEM and TEM images of (**a**,**e**) rGO, (**b**,**f**) srGO (1 h), (**c**,**g**) srGO (2 h), and (**d**,**h**) srGO (3 h).

The morphologies of the rGO and srGO supports were analyzed via a scanning electron microscope (SEM) (Figure 2a–d) and transmission electron microscopy (TEM) (Figure 2e–h). The TEM and SEM images indicated that the change in graphene size depended on the sonication time, from 1 to 3 h. It was confirmed that the size of rGO decreased as the sonication time increased and that graphene sheets reduced from 6.6 to 1.2 μm. The morphologies of the rGO and srGO supports were analyzed via scanning electron microscopy (SEM) (Figure 2a–d) and transmission electron microscopy (TEM) (Figure 2e–h). The TEM and SEM images indicated that the change in the graphene size depended on the sonication time, which ranged from 1 to 3 h. It was confirmed that the size of rGO decreased as the sonication time increased and that graphene sheets were reduced from 6.6 to 1.2 μm in diameter. Graphene that was exfoliated and broken down through the physical sonication process also decreased in size. Brunauer–Emmett–Teller (BET) analysis was performed to examine the changes in the specific surface areas and pore volumes of rGO and srGO, and the results confirmed that the specific surface area and pore volume gradually increased as the sonication time increased (Table 1).

**Figure 3:**
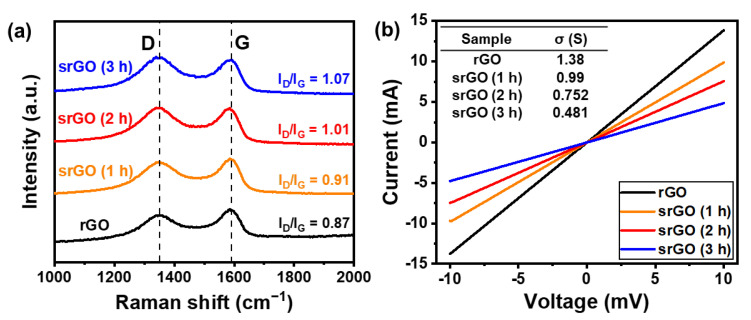
(**a**) Raman spectra and (**b**) representative IV curves of rGO, srGO (1 h), srGO (2 h), and srGO (3 h).

The ratio of defects on the rGO sample surfaces based on the sonication time was confirmed by Raman spectroscopy, as shown in Figure 3a. The G and D bands of the rGO Raman spectrum are located very close to each other, at approximately 1589 and 1334 cm^−1^, respectively. The G band resulted from the vibrational mode of adjacent carbon atoms in the hexagonal structure moving in opposite directions. The D band exhibited crystalline defects that formed during the oxidation and exfoliation of the graphite. The D-band intensity (I_D_)/G-band intensity (I_G_) ratio is a numerical value that is utilized to quantitatively compare the amounts of carbon crystals and defects [29,30]. The Raman spectra confirmed that I_D_/I_G_ increased with sonication time; this is because the D peak increases with the number of dangling defects. The larger the I_D_/I_G_ ratio, the more defects generated during the sonication process. Determining the I_D_/I_G_ value of rGO is an important factor in this process because a sufficient number of defects do not occur if the I_D_/I_G_ value of rGO is too small; therefore, the resulting sample would not have a sufficiently small size nor specific area. If the I_D_/I_G_ value of rGO is too large, excessive defects occur on the graphene surface, thereby reducing the crystallinity of graphene. The conductivity of rGO is also reduced under this condition, which inhibits sufficient electron transfer and greatly reduces the efficiency of the catalyst. 

X-ray photoelectron spectroscopy (XPS) analysis was performed to observe the changes occurring in the surface functional groups as a result of sonication (see Figure S1 and Table 2). The rGO C1s signal has three components corresponding to C–C (284.5 eV), C–O (286.6 eV), and C=O (288.5 eV) functional groups [31]. The I_C–O_/I_C–C_ ratio decreased after 3 h of ultrasonication because of an increase in the surface area and a decrease in the rGO particle size. Oxygen functional groups were introduced into the graphene basal and edge planes during the chemical exfoliation process [32]. Therefore, as the size of rGO decreases, the surface area and number of functional groups of rGO increase. Figure 3b shows the electrical characterization of these samples achieved using a source measure unit (SMU), which obtains current (I)–voltage (V) curves. We found that the electrical conductance of rGO decreased as the sonication time increased from 1 to 3 h. It is well known that graphene has a high in-plane electrical conductance; however, it also has a high contact resistance. As the size of srGO decreased, the contact resistance of bulk rGO increased. Therefore, the electrical conductivity of srGO decreased with increasing sonication time.

**Figure 4:**
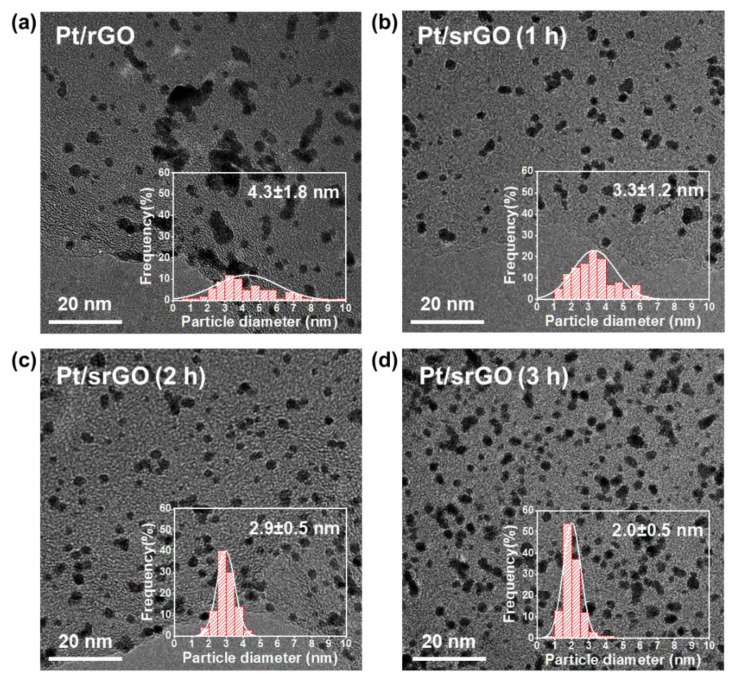
TEM images and corresponding Pt particle size distribution histograms of (**a**) Pt/rGO, (**b**) Pt/srGO (1 h), (**c**) Pt/srGO (2 h), and (**d**) Pt/srGO (3 h).

We utilized the polyol method to synthesize Pt nanoparticles with rGO and various sizes of synthesized srGO supports, and X-ray diffraction (XRD) was performed to determine the effect of the crystalline structure of the Pt nanoparticles on the carbon support (Figure S2a). The XRD patterns of Pt/rGO, Pt/srGO, and Pt/C were apparent on the (111), (200), and (220) planes of the face-centered cubic structure of crystalline Pt located at 2θ = 40°, 46°, and 68° in all samples. Thermogravimetric analysis (TGA) was performed to assess the Pt content in the synthesized catalysts (Figure S2b). These results confirmed that all catalysts contained a consistent Pt content of 20 wt%, indicating that the support activity could be compared to that of Pt of the same weight. The morphologies of the Pt/rGO, Pt/srGO (1 h), Pt/srGO (2 h), and Pt/srGO (3 h) catalysts were compared using TEM (Figure 4a–d). Representative TEM data collected for the Pt/rGO and Pt/srGO samples, as well as the histograms of these samples, were then evaluated. The size of the Pt nanoparticles depended on the size of the support. The average Pt particle size of sonicated rGO decreased from 4.3 ± 1.8 to 3.3 ± 1.2, 2.9 ± 0.5, and finally 2.0 ± 0.5 nm after 1, 2, and 3 h of sonication, respectively. Several obvious agglomerations of Pt nanoparticles with an average size of 4.3 nm were found in Pt/rGO (Figure 4a). In addition, greater aggregation was observed for the Pt/C catalyst (Figure S3). Comparisons of the catalysts with the same metal loading but with increased surface area of the support material revealed that the average particle size of Pt decreased, and the Pt size distribution became narrower. As the size of srGO decreased, the number of oxygen functional groups present in the defects increased, which resulted in an increase in the number of anchoring sites on the rGO surface and a decrease in the Pt nanoparticle size [33]. Therefore, we may infer that a smaller support material results in a narrower Pt size distribution and smaller Pt nanoparticles. 

**Figure 5:**
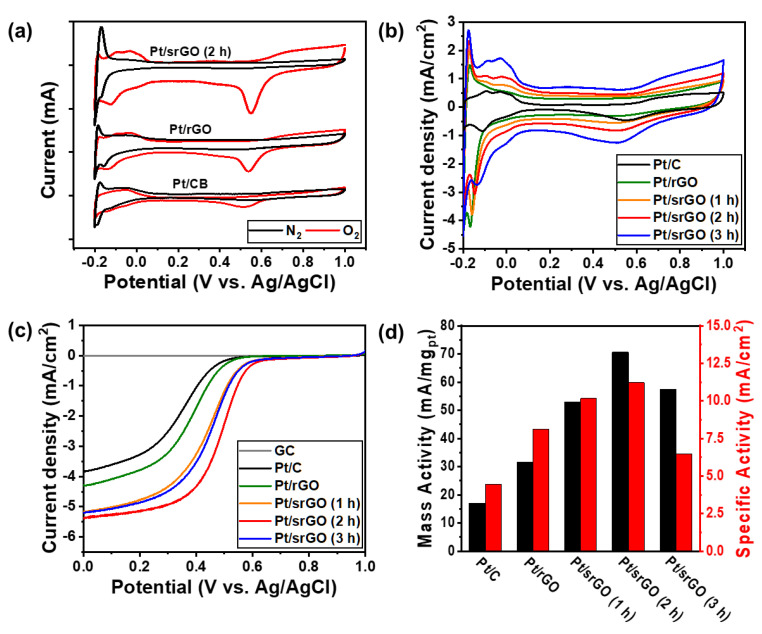
(**a**) CV curves of Pt/rGO, Pt/srGO (2 h), and Pt/C in N_2_-saturated (black lines) and O_2_-saturated (red lines) 0.5 M H_2_SO_4_ at a scan rate of 20 mV/s. (**b**) CV curves of the catalysts in N_2_-saturated 0.5 M H_2_SO_4_ at a scan rate of 20 mV/s. (**c**) Polarization curves of that catalysts in O_2_-saturated 0.5 M H_2_SO_4_ at a scan rate of 5 mV/s and GCE rotational speed of 1600 rpm. (**d**) Mass and specific activities of the catalysts at 0.4 V vs. Ag/AgCl.

To evaluate the fuel cell performance, we conducted linear sweep voltammetry (LSV) and cyclic voltammetry (CV) measurements. Figure 5a shows the typical CV curves of Pt/C, Pt/rGO, and Pt/srGO (2 h), which all exhibit a large cathodic peak at 0.55 V when an O_2_-saturated 0.5 M H_2_SO_4_ solution was the electrolyte; however, this peak was not observed in the N_2_-saturated electrolytes. Figure 5b shows CV curves of the Pt/rGO, Pt/srGO, and Pt/C catalysts, which all consisted of two regions: hydrogen desorption over the range of −0.2 to 0.15 V vs. Ag/AgCl and double layer charging/discharging over the range of 0.15 to 4.0 V vs. Ag/AgCl [34]. These electrolytes were mixed with 99.99% high-purity bubbling N_2_ for 30 min to eliminate O_2_. No significant ORR was observed in the N_2_-saturated electrolytes for all catalysts; however, the ORR was clearly observed with the O_2_-saturated electrolytes (Figure S4a). The electrochemical surface area (ECSA, m^2^/g_Pt_) for hydrogen adsorption can be calculated using the CV curve (Figure 5b), as shown in Equation (1):

(1) $$\mathrm{ECSA}=\frac{S}{2.1\times V\times M},$$

where V is the scan rate (V/s), S represents the integral area between the current and voltage in the hydrogen adsorption area (mA·V), and M is the Pt load (mg). We obtained the ECSAs for the catalysts: Pt/C, Pt/rGO, Pt/srGO (1 h), Pt/srGO (2 h), and Pt/srGO (3 h) (Table 3). Of all the catalysts, Pt/C and Pt/rGO exhibited the lowest ECSAs. Furthermore, for Pt/srGO, the ECSA gradually increased with sonication time. These results clearly show that the size of the Pt nanoparticles decreases, and the areal density increases with the increase in sonication time. The double-layer region mainly reflects the characteristics of srGO, and the current density in the double-layer region increased with sonication time due to the increase in the srGO surface area. Figure 5c shows the polarization curves of Pt/rGO, Pt/srGO, and Pt/C at a rotation speed of 1600 rpm. It can be seen that Pt/srGO (2 h) exhibited a much better onset potential of 575.8 mV vs. Ag/AgCl than Pt/rGO, Pt/srGO (1 h), Pt/srGO (3 h), and Pt/C (Figure S4b). Furthermore, the half-wave potential (E1/2) of Pt/srGO (2 h) was larger than those of Pt/srGO (3 h), Pt/srGO (1 h), Pt/rGO, and Pt/C. Owing to the high electroconductivity and specific area of rGO, the electrochemical properties of Pt/rGO and Pt/srGO were better than those of Pt/C. In addition, we confirmed through the CV curves that a longer sonication time resulted in a larger specific surface area, higher ESCA, and higher current density. Especially, the decrease of catalyst size can increase the active surface area of catalyst particles, which greatly enhanced the ESCA and current density. However, the current density decreased with sonication times longer than 2 h, which indicates that the smaller the size of rGO, the lower its overall electrical conductivity. As shown in Figure 3, the crystallinity of srGO (3 h) is reduced due to excessive surface defects. This results in low electrical conductivity, cannot sufficiently transfer the electrons generated in the electrochemical reaction, thus causing a significant decrease in the efficiency of the catalyst. Therefore, it can be confirmed that too many surface defects are generated in graphene as a result of increased sonication time, which reduces the crystallinity of graphene and the ORR characteristics. We determined the mass activity (MA) and specific activity (SA) of the catalysts to be 0.4 V (vs. Ag/AgCl) based on their LSV curves. The reaction current at 0.4 V was divided by the true surface area and the actual mass of Pt to obtain the SA. The reaction current at 0.4 V was then divided by the actual mass of Pt to obtain the MA. The SA was calculated using Equation (2) as follows:

(2) $$\mathrm{SA}=\frac{I}{{\mathrm{ECSA}\times \mathrm{Pt}}_{\mathrm{Loading}}}$$

Here, Pt_Loading_ represents the loading of the catalyst electrode (mg). The MA was calculated using Equation (3), as shown below:

(3) $$\mathrm{MA}=\frac{I}{{\mathrm{Pt}}_{\mathrm{Loading}}\;}$$

Here, I is the reaction current in the LSV curve at 0.4 V (MA).

The MA and SA shown in Figure 5d were obtained by synchronizing the kinetic current with the mass of Pt and ECSA, respectively. The Pt/srGO (2 h) sample exhibited the best ORR performance with an MA and SA. For comparison, the respective MA and SA of Pt/srGO (3 h), Pt/srGO (1 h), Pt/rGO and Pt/C. Therefore, the optimum conditions for producing a support material with a high specific surface area, low electrical resistance, and high ORR properties are found in graphene treated with hydrochloric acid for 2 h.

**Figure 6:**
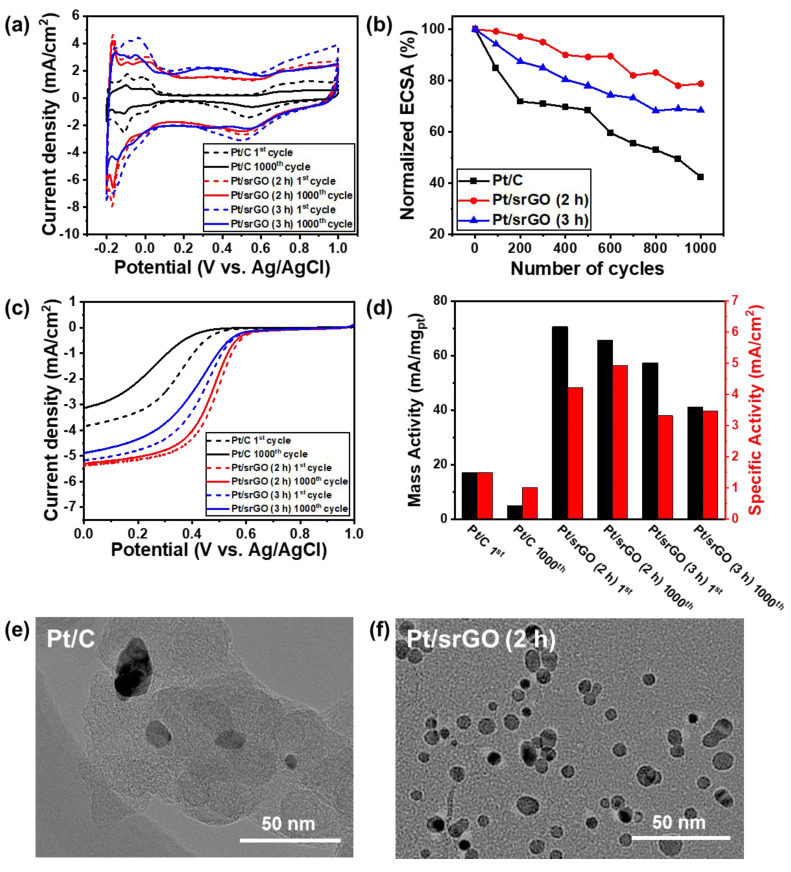
(**a**) CV curves of Pt/srGO (2 h), Pt/srGO (3 h), and Pt/C before and after the ADTs for 1000 cycles in N_2_-saturated 0.5 M H_2_SO_4_ at a scan rate of 50 mV/s. (**b**) ECSAs of the samples over 1000 electrochemical cycles. (**c**) Polarization CV curves of samples before and after ADTs for 1000 cycles in O_2_-saturated 0.5 M H_2_SO_4_ at a scan rate of 5 mV/s and GCE rotational speed of 1600 rpm. (**d**) Mass and specific activities of samples at 0.4 V vs. Ag/AgCl. TEM images of (**e**) Pt/C and (**f**) Pt/srGO (2 h) after ADTs for 1000 cycles.

The stabilities of Pt/CB, Pt/srGO (2 h), and Pt/srGO (3 h) were assessed by performing 1000 cycles of accelerated durability tests (ADTs) from −0.2 to 1.0 V vs. Ag/AgCl. The CV curves were measured in an N_2_-saturated 0.5 M H_2_SO_4_ solution at a scan rate of 50 mV/s before and after the ADT cycles, as shown in Figure 6a. The ECSA was calculated by measuring the coulombic charge for hydrogen desorption in the range of 0.2 to 1.0 V vs. Ag/AgCl. The normalized ESCA, which was evaluated after every 100 cycles, is shown in Figure 6b. The ECSA of Pt/srGO (2 h) decreased by 21.1% after 1000 cycles of ADTs, which is less of a decrease than those observed in the ECSAs of Pt/srGO (3 h) (31.6%) and Pt/C (57.6%). The polarization curves of Pt/srGO (2 h), Pt/srGO (3 h), and Pt/C after 1000 cycles of ADTs are also shown in Figure 6c. The loss of the onset potential of Pt/srGO (2 h) was only 3.8 mV, which is much better than the losses noted for Pt/srGO (9.8 mV) and Pt/C (68 mV). A slightly negative shift of only 16.9 mV (indicating only a 3.39% loss) was observed in the half-wave potential of Pt/srGO (2 h), making this catalyst more effective than Pt/srGO (3 h) and Pt/C whose half-wave potentials negatively shifted by 28.1 mV (indicating a 6.02% loss) and 90.1 mV (indicating a 24.4% loss), respectively. Furthermore, the Pt/srGO (2 h) catalyst experienced a significant decrease in terms of its ESCA, MA, and SA (Figure 6d & Table 4). 

As previously stated, these data were obtained by synchronizing the kinetic current with the mass of Pt and ECSA. The MA of Pt/srGO (2 h) at 0.4 V vs. Ag/AgCl reached 70.6 mA/mg_Pt_, which is much higher than those of Pt/srGO (3 h) and Pt/C. It should be noted that the loss of MA for Pt/srGO (2 h) was only 7.2% after 1000 cycles of ADTs at 0.4 V vs. Ag/AgCl, which was much lower than the losses observed for Pt/srGO (3 h) (28.3%) and Pt/C (71.2%). The Pt/srGO (3 h) catalyst caused a more significant decrease in the ESCA and ORR performance of the system than Pt/srGO (2 h) because the excessive surface defects of the former made srGO vulnerable to carbon oxidation reactions, and its particle bonding was relatively weak due to the presence of smaller particles. The SA of Pt/srGO (2 h) at 0.4 V vs. Ag/AgCl was 2.13 mA/cm^2^, which then increased to 2.49 mA/cm^2^ after the ADTs. For comparison, the SA of Pt/srGO (3 h) (1.67 mA/cm^2^) increased by a smaller amount to only 1.75 mA/cm^2^. This is because the ECSA of Pt/srGO (2 h) showed less of a decrease than that of Pt/srGO (3 h). Additionally, the SA of Pt/C (0.75 mA/cm^2^) decreased to 0.51 mA/cm^2^ because of the rapid decrease in current and its ECSA. The TEM images of Pt/srGO (2 h) and Pt/C were obtained before and after the ADTs. The Pt particles on commercial Pt/C severely agglomerated into large, irregular nanoparticles after the ADTs, as shown in Figure 6e. However, the mean diameter of the Pt nanoparticles on Pt/srGO (2 h) exhibited less aggregation than those on Pt/C after the ADTs, as shown in Figure 6e. Pt/srGO (2 h) was more stable than both Pt/srGO (3 h) and Pt/C. The Pt/C catalyst experienced a significant decrease in ESCA following the ADTs, which was a result of the aggregation of the Pt nanoparticles due to the relatively corrosive nature of carbon [35]. Therefore, Pt/srGO (2h) had the catalyst support with the most suitable defects and Pt nanoparticle size, providing it with the highest durability.

## 3. Materials and Methods

### 3.1. Controlling the Size of rGO

rGO was obtained from Standard Graphene Inc., and strong sonication was used to control its size by breaking the weak carbon bonds at the sites of any defects. First, 150 mg of rGO was mixed with 300 mL of 10 vol% HCl and subjected to 1, 2, or 3 h of sonication. The synthesized srGO samples were then washed with ethanol and deionized water and placed in an oven at 100 °C overnight to dry. 

### 3.2. Synthesizing the Pt/rGO Catalysts

Catalysts were synthesized via the polyol method using a microwave (Multiwave 5000, Anton Paar GmbH, Graz, Austria) and a fixed amount of Pt (20 wt%). First, 80 mg of rGO, srGO, and CB were mixed with 40 mL of ethyl glycol and sonicated for 1 h. A Pt precursor, Chloroplatinic acid solution (H_2_PtCl_6_∙6H2O Sigma-Aldrich), was added to the solution using a magnetic stirrer for approximately 30 min. The pH of the solution was adjusted to 12 using potassium hydroxide. The prepared solution was then heated in a microwave oven. The synthesized catalysts were washed with ethanol and deionized water and placed in a vacuum oven at 50 °C to dry overnight. 

### 3.3. Characterizing the Materials

The surface morphologies and elemental compositions of the samples were analyzed using field-emission SEM (FE-SEM; Hitachi, Tokyo, Japan, SU8020) and TEM (JEOL Ltd., Tokyo, Japan, JEM-2100F). The chemical compositions and functional groups of rGO and srGO were examined via XPS (Thermo Scientific, Waltham, MA, USA, K Alpha+) analysis was then conducted using Al-Kα radiation. The binding energy was normalized according to the position of the C 1s peak as a result of the adsorbed hydrocarbon fragments. Raman spectroscopy (WITec, Ulm, Germany, alpha300s) was conducted to identify any changes in the defects within rGO and srGO using a DXR Raman microscope with incident light at a wavelength of 532 nm. We analyzed the extent of crystallinity using XRD (Rigaku, Tokyo, Japan, Ultima IV/Rigaku) with Cu Kα (λ = 0.15406 nm) radiation over the 2θ range of 10° to 85° at a scan rate of 1°/min. The electrical characteristics of rGO and srGO were measured with a SMU (Keithley, Tektronix, 2400) in a pressure cell (M&S Vacuum) under uniaxial pressure (5 MPa) at room temperature. The Pt nanoparticle contents of the prepared catalysts were measured using TGA (Mettler Toledo, Greifensee, Switzerland, TGA/DSC1) under air flow at temperatures ranging from 3 to 200 °C, which were maintained for 2 h and subsequently increased to 850 °C at a rate of 10 °C/min.

### 3.4. Measuring the Electrochemical Properties

Electrochemical measurements of the catalysts were performed using a half-cell system with three electrodes (BioLogic, Seyssinet-Pariset, France, VSP 300). A Pt wire and Ag/AgCl electrode were used as the counter and reference electrodes, respectively. The catalyst was coated on the surface of glassy carbon (GC) (AFE5T050GC, 5.0 mm disk OD, Pine Research, Durham, USA) and used as the working electrode. To measure the electrochemical activity of the catalyst, approximately 5 mg of a mixture containing IPA (1 mL) and 5 wt% Nafion solution (60 µL) was added and sonicated for 30 min until a homogenous suspension was obtained. The GC electrode was prepared by drop-casting 20 uL of this solution to obtain a catalytic suspension using a micropipette, followed by drying at 27 °C for 20 min. CV curves were measured at a scan rate of 20 mV/s within a range of −0.2 to 1.0 V in N_2_-saturated 0.5 M H_2_SO_4_. LSV measurements with a scan rate of 5 mV/s were obtained with RDE rotation rates of 1600 rpm in O_2_-saturated 0.5 M H_2_SO_4_. ADTs were performed at a scan rate of 20 mV/s for 1000 cycles from 0.2 to 1.0 V vs. Ag/AgCl in 0.5 M H_2_SO_4_.

## 4. Conclusions

We demonstrated highly efficient oxygen reduction catalysts composed of uniform Pt nanoparticles on srGO. The rGO size was controlled by applying a physical force via ultrasonication, where increased sonication times decreased the rGO size. The srGO enabled the morphological control of the Pt nanoparticles, and the resultant, prepared catalysts provided efficient surface reactions and exhibited large surface areas and high metal dispersions. The Pt/srGO catalysts prepared with 2 h of sonication exhibited excellent oxygen reduction performances and high stability over 1000 cycles of ADTs. The detailed analysis of the structural and electrochemical properties of the resulting catalysts suggested that both the chemical functionality and electrical conductivity greatly contribute to their enhanced efficiency. We believe that our study will make a significant contribution to the currently existing literature and inspire future studies on the electrochemical applications of these materials owing to their versatility regarding various types of fuel cells.

## Figures and Tables

**Table 1:** BET surface area, pore volume, and pore size of rGO and srGO.

Sample	SBET (m^2^/g)	Pore Volume (cm^3^/g)	Pore Size (nm)
rGO	372.9	1.36	14.4
srGO (1 h)	402.7	1.43	14.1
srGO (2 h)	427.0	1.52	13.9
srGO (3 h)	441.0	1.57	14.3

**Table 2:** C/O ratios of rGO and srGO calculated from XPS data.

Sample	C Bonding	O Bonding	C/O Ratio
rGO	93.33%	6.66%	14.01
srGO (1 h)	91.9%	8.11%	11.33
srGO (2 h)	89.78%	8.49%	10.57
srGO (3 h)	88.04%	10.36%	8.50

**Table 3:** ECSA, E_onset_, E_half-wave_, Ma and SA of the catalysts.

Sample	ECSA (m^2^/g_Pt_)	E_onset_ (mV)	E_half-wave_ (mV)	MA (mA/mg_pt_)	SA (mA/cm^2^)
Pt/C	38.78	467.9	368.9	17.1	2.25
Pt/rGO	39.48	493.8	399.8	31.7	4.09
Pt/srGO (1 h)	52.82	548.0	457.8	53.1	5.12
Pt/srGO (2 h)	63.69	575.8	497.8	70.6	5.66
Pt/srGO (3 h)	89.88	552.8	466.9	57.4	3.25

**Table 4:** ECSA, E_onset_, E_half-wave_, MA and SA change of the catalysts during durability analysis.

Sample	As-Prepared					After Durability Analysis				
	ECSA (m^2^/g_Pt_)	E_onset_ (mV)	E_half-wave_ (mV)	MA (mA/mg_pt_)	SA (mA/cm^2^)	ECSA (m^2^/g_Pt_)	E_onset_ (mV)	E_half-wave_ (mV)	MA (mA/mg_pt_)	SA (mA/cm^2^)
Pt/C	115.5	467.9	368.9	17.1	0.75	48.99	399.9	278.8	4.92	0.51
Pt/srGO (2 h)	169.3	575.8	497.8	70.6	2.13	134.5	572.0	480.9	65.6	2.49
Pt/srGO (3 h)	174.8	552.8	466.9	57.4	1.67	119.7	543.0	438.8	14.2	1.75

## Data Availability

The data that support the findings of this study are available from the corresponding authors upon reasonable request.

## Supplementary Materials

The following are available online at https://www.mdpi.com/article/10.3390/ijms222212300/s1, Figure S1: The C 1s region of the X-ray photoelectron spectroscopy (XPS) spectra. Figure S2: X-ray diffraction (XRD) patterns and thermogravimetric analysis (TGA) curves of the catalyst samples. Figure S3: Scanning electron microscopy (SEM) image of Pt/C. Figure S4: Cyclic voltammetry (CV) curves of the catalyst samples and onset potentials in the polarization curves of the catalyst samples.

## References

[1] Cha B.-C., Jun S., Jeong B., Ezazi M., Kwon G., Kim D., Lee D.H. (2018). Carbon nanotubes as durable catalyst supports for oxygen reduction electrode of proton exchange membrane fuel cells. J. Power Sources.

[2] Sharma S., Pollet B.G. (2012). Support materials for PEMFC and DMFC electrocatalysts—A review. J. Power Sources.

[3] Lee D.H., Lee W.J., Kim S.O., Kim Y.-H. (2011). Theory, Synthesis, and Oxygen Reduction Catalysis of Fe-Porphyrin-Like Carbon Nanotube. Phys. Rev. Lett..

[4] Wu J., Yuan X.Z., Martin J.J., Wang H., Zhang J., Shen J., Wu S., Merida W. (2008). A review of PEM fuel cell durability: Degradation mechanisms and mitigation strategies. J. Power Sources.

[5] Service R.F. (2002). Shrinking Fuel Cells Promise Power in Your Pocket. Science.

[6] Okada M., Konta Y., Nakagawa N. (2008). Carbon nano-fiber interlayer that provides high catalyst utilization in direct methanol fuel cell. J. Power Sources.

[7] Kim J., Kim S.-I., Jo S.G., Hong N.E., Ye B., Lee S., Dow H.S., Lee D.H., Lee J.W. (2020). Enhanced activity and durability of Pt nanoparticles supported on reduced graphene oxide for oxygen reduction catalysts of proton exchange membrane fuel cells. Catal. Today.

[8] Li Z., Gao Q., Zhang H., Tian W., Tan Y., Qian W., Liu Z. (2017). Low content Pt nanoparticles anchored on N-doped reduced graphene oxide with high and stable electrocatalytic activity for oxygen reduction reaction. Sci. Rep..

[9] Lee D.H., Kim J.E., Han T.H., Hwang J.W., Jeon S., Choi S.-Y., Hong S.H., Lee W.J., Ruoff R.S., Kim S.O. (2010). Versatile Carbon Hybrid Films Composed of Vertical Carbon Nanotubes Grown on Mechanically Compliant Graphene Films. Adv. Mater..

[10] Lee S.H., Lee D.H., Lee W.J., Kim S.O. (2011). Tailored Assembly of Carbon Nanotubes and Graphene. Adv. Funct. Mater..

[11] Hong J.-Y., Lee E., Jang J. (2013). Electro-responsive and dielectric characteristics of graphene sheets decorated with TiO2nanorods. J. Mater. Chem. A.

[12] Yin J., Wang X., Chang R., Zhao X. (2012). Polyaniline decorated graphene sheet suspension with enhanced electrorheology. Soft Matter.

[13] Zhang W.L., Liu Y.D., Choi H.J., Kim S.G. (2012). Electrorheology of Graphene Oxide. ACS Appl. Mater. Interfaces.

[14] Zhang W.L., Choi H.J. (2011). Fast and Facile Fabrication of a Graphene Oxide/Titania Nanocomposite and Its Electro-Responsive Characteristics. Chem. Commun..

[15] He D., Cheng K., Peng T., Sun X., Pan M., Mu S. (2012). Bifunctional effect of reduced graphene oxides to support active metal nanoparticles for oxygen reduction reaction and stability. J. Mater. Chem..

[16] Ye B., Lee M., Jeong B., Kim J., Lee D.H., Baik J.M., Kim H.-D. (2019). Partially reduced graphene oxide as a support of Mn-Ce/TiO2 catalyst for selective catalytic reduction of NOx with NH3. Cat. Today.

[17] Antolini E. (2012). Graphene as a new carbon support for low-temperature fuel cell catalysts. Appl. Catal. B Environ..

[18] Lee M., Kim S.-I., Lee M.-J., Ye B., Kim T., Kim H.-D., Lee J., Lee D. (2021). Effect of Catalyst Crystallinity on V-Based Selective Catalytic Reduction with Ammonia. Nanomaterials.

[19] Pushkareva I., Pushkarev A., Kalinichenko V., Chumakov R., Soloviev M., Liang Y., Millet P., Grigoriev S. (2021). Reduced Graphene Oxide-Supported Pt-Based Catalysts for PEM Fuel Cells with Enhanced Activity and Stability. Catalysts.

[20] Yadav R., Subhash A., Chemmenchery N., Kandasubramanian B. (2018). Graphene and Graphene Oxide for Fuel Cell Technology. Ind. Eng. Chem. Res..

[21] He D., Kou Z., Xiong Y., Cheng K., Chen X., Pan M., Mu S. (2014). Simultaneous sulfonation and reduction of graphene oxide as highly efficient supports for metal nanocatalysts. Carbon.

[22] Baamran K.S., Tahir M., Mohamed M., Khoja A.H. (2020). Effect of support size for stimulating hydrogen production in phenol steam reforming using Ni-embedded TiO2 nanocatalyst. J. Environ. Chem. Eng..

[23] Luo S., Barrio L., Nguyen-Phan T.-D., Vovchok D., Johnston-Peck A.C., Xu W., Stach E.A., Rodriguez J.A., Senanayake S.D. (2017). Importance of Low Dimensional CeOx Nanostructures in Pt/CeOx–TiO_2_ Catalysts for the Water–Gas Shift Reaction. J. Phys. Chem. C.

[24] Ferencz Z., Erdohelyi A., Baán K., Oszkó A., Óvári L., Kónya Z., Papp C., Steinruck H.-P., Kiss J. (2014). Effects of support and Rh additive on Co-based catalysts in the ethanol steam reforming reaction. ACS Catal..

[25] Kočí K., Obalová L., Matějová L., Plachá D., Lacný Z., Jirkovský J., Solcova O. (2009). Effect of TiO_2_ particle size on the photocatalytic reduction of CO_2_. Appl. Catal. B.

[26] Soykal I.I., Sohn H., Ozkan U.S. (2012). Effect of Support Particle Size in Steam Reforming of Ethanol over Co/CeO_2_ Catalysts. ACS Catal..

[27] Haga F., Nakajima T., Yamashita K., Mishima S. (1997). Effect of particle size on steam reforming of ethanol over alumina-supported cobalt catalyst. Nippon. Kagaku Kaishi.

[28] Aramouni N.A.K., Zeaiter J., Kwapinski W., Ahmad M.N. (2017). Thermodynamic analysis of methane dry reforming: Effect of the catalyst particle size on carbon formation. Energy Convers. Manag..

[29] Malard L.M., Pimenta M.A., Dresselhaus G., Dresselhaus M.S. (2009). Raman spectroscopy in graphene. Phys. Rep..

[30] Ye B., Kim S.-I., Lee M., Ezazi M., Kim H.-D., Kwon G., Lee D.H. (2020). Synthesis of oxygen functionalized carbon nanotubes and their application for selective catalytic reduction of NOx with NH3. RSC Adv..

[31] Shin K.-Y., Hong J.-Y., Lee S., Jang J. (2012). Evaluation of anti-scratch properties of graphene oxide/polypropylene nanocomposites. J. Mater. Chem..

[32] Shin K.-Y., Lee S., Hong S., Jang J. (2014). Graphene Size Control via a Mechanochemical Method and Electro-Responsive Properties. ACS Appl. Mater. Interfaces.

[33] Eslava J.L., Sun X., Gascon J., Kapteijn F., Rodríguez-Ramos I. (2017). Ruthenium particle size and cesium promotion effects in Fischer-Tropsch synthesis over high-surface-area graphite supported catalysts. Catal. Sci. Technol..

[34] Guo S., Zhang S., Su D., Sun S. (2013). Seed-mediated synthesis of core/shell FePtM/FePt (M = Pd, Au) nanowires and their electrocatalysis for oxygen reduction reaction. J. Am. Chem. Soc..

[35] Guo L., Jiang W.-J., Zhang Y., Hu J.-S., Wei Z.-D., Wan L.-J. (2015). Embedding Pt Nanocrystals in N-Doped Porous Carbon/Carbon Nanotubes toward Highly Stable Electrocatalysts for the Oxygen Reduction Reaction. ACS Catal..

