# Supplementary material for: Small Reduced Graphene Oxides for Highly Efficient Oxygen Reduction Catalysts
Source: Int J Mol Sci. 2021 Nov 14;22(22):12300. doi: 10.3390/ijms222212300 (PMC8625579; doi:10.3390/ijms222212300)
Supplement: Supplementary file 1 [file ijms-22-12300-s001.zip › ijms-1449203-supplementary.pdf]

Small Reduced Graphene Oxides for Highly Efficient Oxygen Reduction Catalysts

Su-Jeong Bak [1,2,†], Sun-I Kim [1,†], Su-yeong Lim [1,2], Taehyo Kim [1], Se-Hun Kwon [2,*], Duck Hyun Lee [1,*]

[1] Green Materials and Processes R&D Group, Korea Institute of Industrial Technology, Ulsan, 44413, Republic of Korea;

baksj@kitech.re.kr (S.-J.B.); sunikim@kitech.re.kr (S.-I.K.); swimmingly@kitech.re.kr (S.L.); thkim0215@kitech.re.kr (T.K.)

[2] Department of Materials Science & Engineering, Pusan National University, Busan, Republic of Korea

* Correspondence: dulee@kitech.re.kr (D.H.L.); sehun@pusan.ac.kr (S.-H.K.);

Tel: +82-52-980-6709 (D.H.L.); +82-51-3775 (S.-H.K.)

† These authors contributed equally to this work.

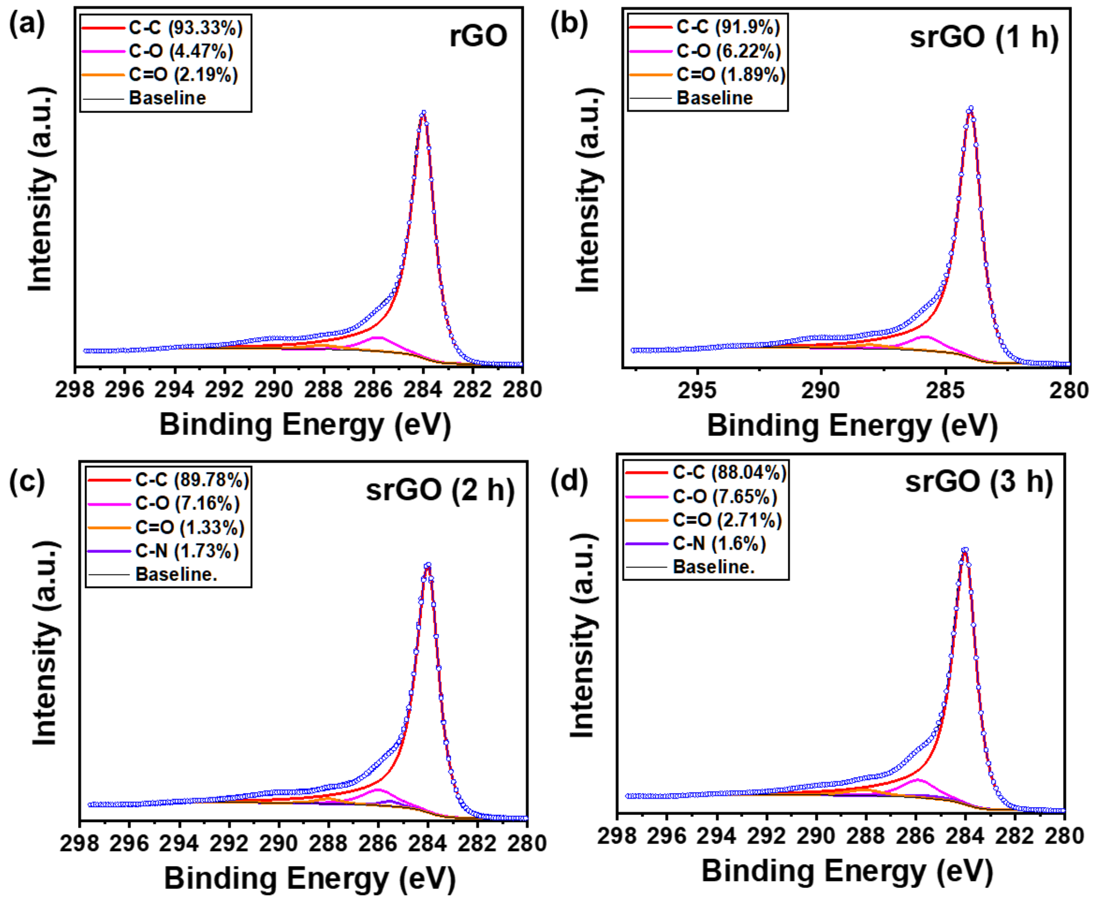

Figure S1. The C 1s region of the X-ray photoelectron spectroscopy (XPS) spectra of (a) reduced graphene oxide (rGO), (b) small rGO (srGO) (1 h), (c) srGO (2 h), and (d) srGO (3 h).

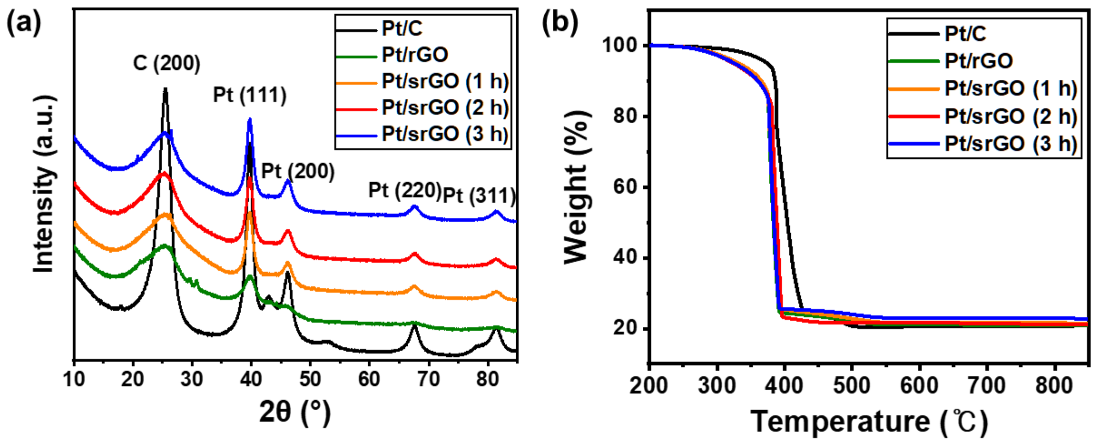

Figure S2. (a) X-ray diffraction (XRD) patterns and (b) thermogravimetric analysis (TGA) curves of the catalyst samples.

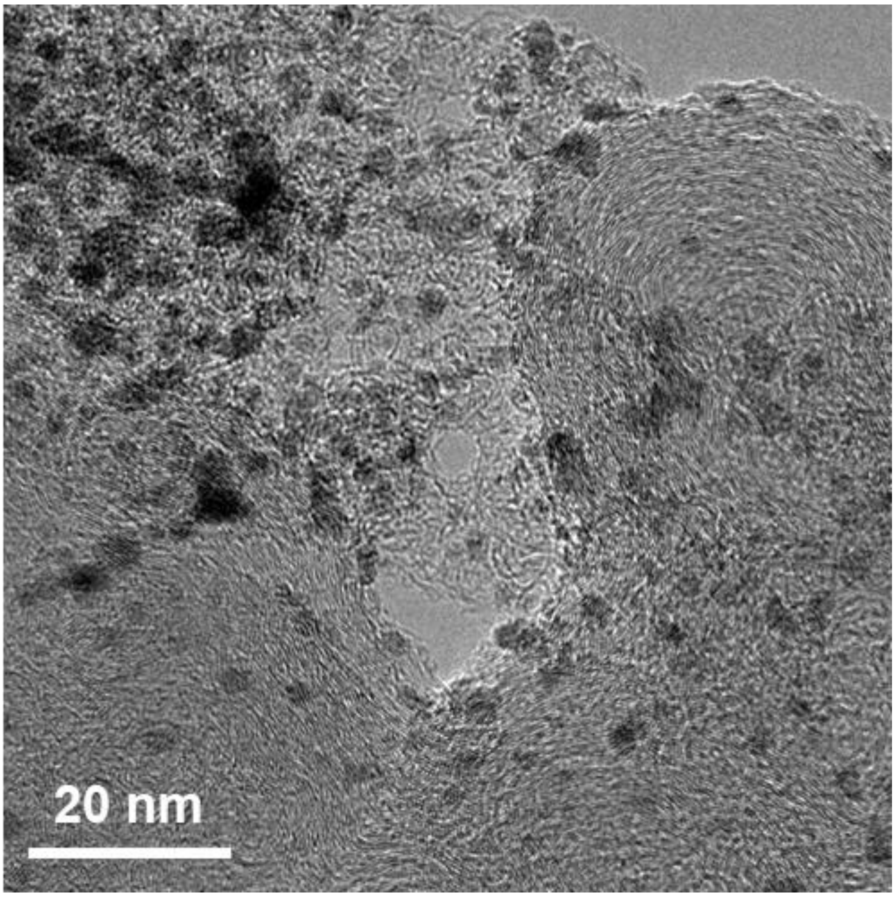

Figure S3. Scanning electron microscopy (SEM) image of Pt/C synthesized using the polyol process.

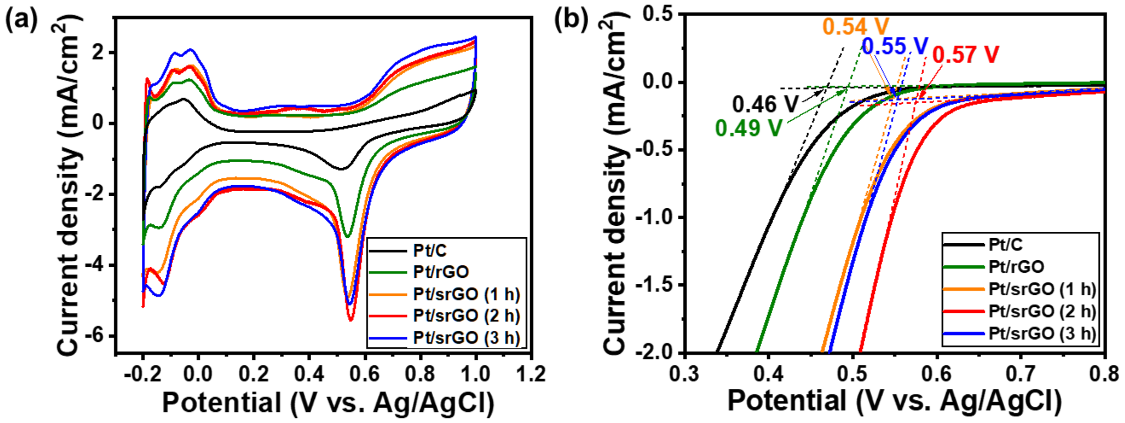

Figure S4. (a) Cyclic voltammetry (CV) curves of the catalyst samples in O_2-saturated 0.5 M H_2SO_4 collected at a scan rate of 20 mV/s. (b) Onset potentials in the polarization curves of the catalyst samples in O_2-saturated 0.5 M H_2SO_4 collected at a scan rate of 5 mV/s and a glassy carbon electrode rotational speed of 1,600 rpm.
